# A Novel Bispecific Anti-IL17/VEGF Fusion Trap Exhibits Potent and Long-Lasting Inhibitory Effects on the Development of Age-Related Macular Degeneration

**DOI:** 10.1155/bri/1405338

**Published:** 2024-12-21

**Authors:** Lan Deng, Lihua Wang, Yun Meng, Jidai Zheng, Xia Dong, Ying Chen, Haomin Huang

**Affiliations:** Development of Research and Development, Sunshine Guojian Pharmaceutical (Shanghai) Co. Ltd., a 3SBio Inc. Company, 399 Libing Road, Shanghai 201203, China

## Abstract

Age-related macular degeneration (AMD) is a severe eye disease in people aged 60 years and older. Although anti-VEGF therapies are effective in treating neovascular AMD (NvAMD) in the clinic, up to 60% of patients do not completely respond to the therapies. Recent studies have shown that blood-derived macrophages and their associated proinflammatory cytokines may play important roles in the development of persistent disease and resistance to anti-VEGF therapy. To address this issue, we constructed an antibody-based bispecific fusion protein that can simultaneously inhibit IL-17-induced inflammation and VEGF-mediated neovascularization. As a result, the bispecific fusion protein 17V05 effectively inhibited multiple proinflammatory cytokines and chemokines, as well as laser-induced choroidal neovascularization (CNV). More importantly, 17V05 also exhibited stronger and longer inhibitory effects than conbercept in vivo. Thus, we provide a novel and promising strategy for treating AMD patients who are not sensitive to anti-VEGF therapies.

## 1. Introduction

As the aging population worldwide is exponentially increasing, age-related macular degeneration (AMD), an eye disease that seriously threatens people older than 60 years [1, 2], has become the most common cause of blindness globally [3, 4]. The number of people with AMD is expected to increase to 196 million by 2020 and 288 million by 2040 [5].

Late-stage AMD results in loss of central visual acuity, leading to severe and permanent visual impairment, and is often characterized as one of the two types, neovascular AMD (NvAMD) (also known as wet or exudative AMD) or non-neovascular AMD (also known as dry, atrophic, or nonexudative AMD) [1]. NvAMD is characterized by choroidal neovascularization (CNV) with lesions, such as the presence of fluid or retinal hemorrhage, retinal pigment epithelial (RPE) detachments, and hard exudate, resulting in rapid and severe vison loss [1, 6]. Vascular endothelial growth factor (VEGF) inhibitors, including bevacizumab, ranibizumab, brolucizumab, conbercept, and aflibercept, which can effectively inhibit the development of new blood vessels, have been widely used to successfully treat NvAMD for decades. However, 20%–60% of patients do not respond to anti-VEGF therapies completely with some syndromes, such as persistent disease activity (PDA), which is characterized as persistent fluid exudation, unresolved or new hemorrhage, and/or progressive lesion fibrosis, as well as suboptimal vision recovery (SVR), which is defined as a failure to achieve functional visual acuity of 20/40 or better [7–11]. Capillary CNV is highly responsive to anti-VEGF therapies and rarely exhibits PDA, whereas high-flow CNV forms lesions with arteriolarization and perivascular fibrosis and thus tends to develop PDA through a mechanism called neovascular remodeling (NVR), in which blood-derived macrophages play an important role [11]. Thus, the pathobiology of arteriolar CNV is believed to be distinct from that of capillary CNV, where angiogenesis mediated by VEGF and regulated by angiopoietin/Tie2, platelet-derived growth factor (PDGF), transforming growth factor-*β* (TGF*β*), etc. plays a key role [12–15]. As a result, arteriolar CNV is refractory to anti-VEGF therapy and urgently requires a novel therapeutic strategy for its cure.

Inflammation is one of the hallmarks of AMD, and proinflammatory cytokines are closely associated with the pathogenesis of this disease [16–19]. Among a number of proinflammatory cytokines, growing evidence has shown that IL-17A, in particular, may play a central role in the pathogenesis of AMD [20]: (1) IL-17A and the associated genes in its signaling pathways were significantly upregulated in AMD patients [21, 22]; (2) IL-17A activated NLRP3 inflammasomes, which is believed to be a key factor for the cause of AMD, and thus promoted the production of IL-1*β*, a master proinflammatory cytokine in response to reactive oxygen species [23]; (3) IL-17A induced choroidal endothelial cell proliferation and migration as well as the formation of capillary-like structures [24, 25]; (4) IL-17A may recruit granulocytes to the inflammatory lesions of AMD, leading to the development of PDA [11, 26]; and (5) both IL-17A receptors, IL-17RA and IL-17RC, are expressed in human primary RPE cells, and the epigenetic modification of the receptor, such as methylation of IL-17RC, is closely associated with RPE cell degeneration and CNV in vitro [27]. Given the above roles of IL-17 in the pathogenesis of ocular neovascular diseases, targeting IL-17 may be a promising approach for treating AMD, especially for patients with PDA [28, 29]. Indeed, neutralization of IL-17A with an anti-IL-17A antibody was able to mitigate M1 macrophage polarization and attenuate retinal and CNV in animal models [30].

In this study, we constructed an antibody-based bispecific fusion protein that can simultaneously inhibit IL-17-induced inflammation and VEGF-mediated neovascularization. As a result, the bispecific fusion protein 17V05 effectively inhibited multiple proinflammatory cytokines and chemokines, including IL-6, IL-8, CXCL1, and MCP-1, which are all involved in the development of AMD [17]. In vivo, 17V05 was more potent in the inhibition of laser-induced CNV than aflibercept, a marketed VEGF inhibitor currently used as the standard of care for AMD, and exhibited stronger and longer inhibitory effects in a persistent retinal neovascularization (RNV) model [31] than conbercept, another marketed VEGF inhibitor. Thus, we provide a novel and promising strategy for treating AMD patients with PDAs.

## 2. Materials and Methods

### 2.1. Cell Culture

The RPE cell line ARPE-19 (Nanjing Cobioer BioSciences Company, Cat#: CBP60759, Nanjing, China) was cultured in DMEM/F12 (Gibco, Cat#: 11,330-032, Hong Kong, China) with 10% FBS and 1% penicillin/streptomycin in a humidified CO_2_ incubator at 37°C. FreeStyle 293 F cells (Thermo Fisher Scientific, Cat#: R79007, Hong Kong, China) were cultured in FreeStyle 293 medium (Gibco, Cat#: 12338018) with 1% penicillin/streptomycin. KDR/NFAT-RE HEK293 cells (Promega, Cat#: GA2001, Beijing, China) were cultured in DMEM (Gibco, Cat#: 10569010) with 10% FBS and 1% penicillin/streptomycin.

### 2.2. Inhibition of VEGF and Cytokine Secretion Assays

ARPE-19 cells in logarithmic growth phase were collected and suspended in DMEM/F12 medium (without FBS), and the cell density was adjusted to 1.5 e5/mL and inoculated into 96-well plates with 100 *μ*L of cell suspension per well overnight. After cell attachment, the supernatant was discarded and 50 *μ*L of complete medium (DMEM/F12 + 10% FBS + 1% PS) with IL-17A (final concentration 100 ng/mL) and 50 *μ*L of the gradient-diluted antibody to be tested were added to each well. After 24 h of co-incubation, VEGF level in the cell culture supernatant was determined using the VEGF165 Matched ELISA Human Antibody Pair Set (SinoBiological, Cat#: SEK11066, Beijing, China).

In the case of cytokine secretion assays, cell culture methods were the same as above except that the co-incubation time of cells with IL-17A and antibodies was 24 h for IL-6, IL-8, and CXCL1 and 48 h for MCP-1. Cytokine levels in the cell culture supernatant were determined using the appropriate kits: IL-6, IL-8, CXCL1, and MCP-1 were measured separately using the IL-6 Matched ELISA Human Antibody Pair Set (SinoBiological, Cat#: SEKB10395); IL-8/CXCL8 Matched ELISA Human Antibody Pair Set (SinoBiological, Cat#: SEK10098); CXCL1 Matched ELISA Human Antibody Pair Set (SinoBiological, Cat#: SEK10877); and MCP-1/CCL2 Matched ELISA Human Antibody Pair Set (SinoBiological, Cat#: SEK10134).

### 2.3. KDR/NFAT-RE Cell–Based Assay

KDR/NFAT-RE HEK293 cells (Promega, Cat#: GA2001) were engineered to express Luc2P under the control of the NFAT response element, as well as exogenous KDRs. Assays were performed according to the manufacturer's instructions. Briefly, KDR/NFAT-RE cells were seeded at 4 × 10^4^ cells/well in 50 *μ*L in white 96-well plates. Then, 25 *μ*L of 3x serially diluted antibodies in DMEM containing 10% FBS and 30 ng/mL VEGF was added to the plates and incubated in a 37°C humidified incubator with 5% CO_2_ for 6 h. Then, 75 *μ*L of Bio-Glo Reagent was added to each well, and the plates were incubated at room temperature for 5–30 min. Luminescence was measured on a SpectraMax i3x.

### 2.4. Mouse Model of CNV and Fundus Fluorescein Angiography (FFA) Analysis

Animal care and in vivo experiments were approved by the IACUC of PharmaLegacy Laboratories (Shanghai) Co. Ltd. and performed according to approved protocols (approval code: PL21-1132-2). All methods were performed in accordance with the relevant guidelines and regulations, including the CO2 euthanasia method. Briefly, at the end of the experiments, animals were placed in a ventilation cage in batch, and CO2 gas was slowly passed through at a rate to ensure 20% of the gas in the box per minute, while observing the state of the animals. Gas supply was maintained for at least 1 min after apparent clinical death to reduce the possibility of possible resuscitation of the animals. After animals completely lost breathing and heartbeat, and cervical dislocation was applied to ensure the death of the animals. The study is reported in accordance with ARRIVE guidelines. CNV was induced by laser photocoagulation with rupture of Bruch's membrane. Ten-week-old female B-hIL17A mice (mice expressing the human IL-17A, purchased from BIOCYTOGEN, MA, USA, *n* = 8) were anesthetized with ketamine hydrochloride (100 mg/kg body weight), and their pupils were dilated with 1% tropicamide. The 532 nm laser was used to photocoagulate 3 points at equal distances around the optic disc at 1.5-2 PD from the optic disc in both eyes, cauterizing 4 laser spots, which should be positioned to avoid the large retinal blood vessels. On Day 7 after photocoagulation, mice were randomized into 3 groups and given 2 *μ*L of PBS (control group) or 87 *μ*M of 17V05 or aflibercept per eye. On Day 14, FFA was performed before sacrifice. After being anesthetized, the pupils of the mice were dilated, and 2% fluorescein sodium (Fluorescite; Alcon, Tokyo, Japan) was injected intraperitoneally. FFA images were obtained at intervals of 2 min. The FFA images were analyzed using ImageJ software (NIH, United States). As for isolectin IB4 staining, the eyes of mice were fixed in 4% formalin followed by staining with 10 μg/mL isolectin IB4, DyLight 649 (VectorLabs, Cat#: DL-1208, CA, USA).

For the measurement of leakage areas, a built-in software program of the Heidelberg Spectralis instrument was used, using FFA images with a score of 3 (see below for the scoring criteria). In the case of isolectin IB4 immunofluorescence staining, lesion areas were measured by co-staining with isolectin IB4 and DAPI on RPE/choroid flat mount. Fluorescent images were taken by EVOS FL Imaging System, ImageJ software was used to quantify CNV lesion areas (Table 1).

### 2.5. Persistent RNV Model

Animal care and in vivo experiments were approved by the IACUC of Sunshine Guojian Pharmaceutical (Shanghai) Co. Ltd. and performed according to approved protocols (approval code: AS-2022-020). At the end of the experiments, CO2 euthanasia method was applied as described above. The study is reported in accordance with ARRIVE guidelines. After SD rats (Vital River, Beijing, China, all females *n* = 8) were anesthetized by an intraperitoneal injection of 10 mL/kg with a mixture of Zoletil50 (Virbac, Carros, France) and Sumianxin II (Military Veterinary Institute, China) at a ratio of 10:1, a single intravitreal injection in each eye with 2 *μ*L of 100 mg/mL DL-*α*-AAA (Sigma, Cat#: A0637, Beijing, China) was performed to induce persistent RNV (Day 0). After one week (on Day 7), the rats were randomized into 3 groups. The control group was administered 5 *μ*L of PBS, and the experimental groups were administered 5 *μ*L of conbercept or XAB4-D2 at equimolar concentrations (87 *μ*M). On Day 14 and Day 21, FFA images were taken and then graded according to fluorescence leakage.

### 2.6. Statistical Analysis

IC_50_ and EC_50_ values were determined using GraphPad Prism 9 (GraphPad Software). Unless otherwise noted, all numerical data were presented as mean ± standard deviation (SD). *p* values were calculated using a two-way ANOVA multiple comparison test. In all tests, differences with *p* values < 0.05(^∗^) were considered statistically significant.

## 3. Results

### 3.1. The Construction of the Bispecific Fusion Protein 17V05

**Figure 1:**
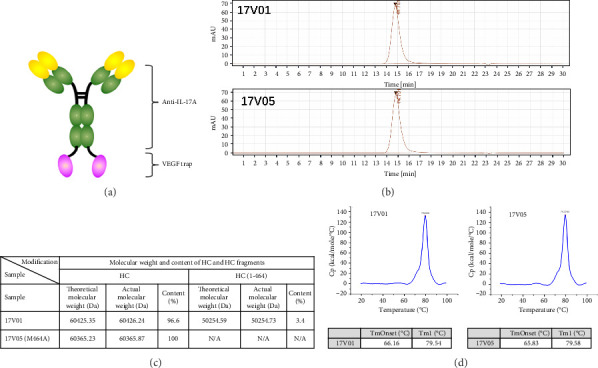
The construction of the bispecific fusion protein 17V05. (a) Schematics of the 17V05 fusion protein structure. (b) SEC showed that both 17V01 and 17V05 have monomeric contents over 95% after one-step affinity purification. (c) Mass spectrometry showed that there is cleavage between M464/Y465 in D2 of the bispecific fusion trap. The 17V05 protein with an M464A mutation prevented cleavage at this site. (d) DSC showed that the *T*_onsets_ of 17V01 and 17V05 were 66.16°C and 65.83°C and the *T*_*m*_1 values were 79.54°C and 79.58°C, respectively. DSC: differential scanning calorimetry, *T*_onsets_: melting temperature at onset, *T*_*m*_1: melting temperature peak 1, HC: heavy chain, and Da: Dalton.

We fused domain 2 of VEGF receptor 1 (D2) to the C-terminus of the heavy chain of a clinical stage anti-IL-17A monoclonal antibody (mAb) 608, developed by Sunshine Guojian Pharmaceutical, via a (G4S) × 3 linker and co-expressed the modified heavy chain with the light chain of 608 (Figure 1(a)). The resultant 17V01 was expressed in 293HEK cells and mostly monomeric (95% above) after one-step purification with protein A chromatography (Figure 1(b)). However, further characterization in a heat-acceleration assay showed that 17V01 was not stable, as the amount of a breakdown product gradually increased during the prolonged heat challenge at 37°C for up to a month. Mass spectrometry showed that the clipping resulted from a break between M464/Y465 in D2. We then made multiple variants with mutations at M464 via site-directed mutagenesis and found that a variant carrying the M464A mutation was stable throughout the heat acceleration assay with no clippings (Figure 1(c)). The variant 17V05 exhibited favorable physicochemical profiles in size-exclusion chromatography (SEC) and differential scanning calorimetry (DSC) and was selected for further analysis (Figures 1(b) and 1(d)).

### 3.2. The Fusion Protein 17V05 Simultaneously Bound to IL-17A and VEGF

**Figure 2:**
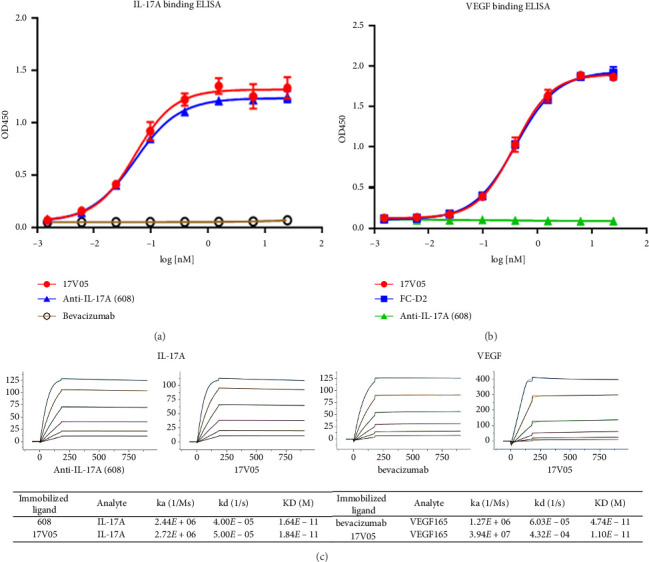
The 17V05 protein simultaneously bound to IL-17A and VEGF as strongly as its parental mAbs. (a) The binding affinity of 17V05 for IL-17A was measured in duplicate by ELISAs and compared to that of the parental anti-IL17A mAb 608. Bevacizumab (Avastin) was used as a negative control (*n* = 2). (b) The ability of 17V05 to bind to VEGF was examined in duplicate by ELISAs and compared to that of FC-D2 (D2 was fused to the N-terminus of an Fc). mAb 608 was used as a negative control (*n* = 2). (c) The equilibrium dissociation constants (KDs) of 17V05 were obtained and compared to those of the parental mAbs 608 and bevacizumab (*n* = 3). *N* = the number of independent experiments. ELISA: enzyme-linked immunosorbent assay; KD: the equilibrium dissociation constant; Ka: the association constant; Kd: the dissociation constant.

We next examined the ability of 17V05 to bind to its targets IL-17A and VEGF. The ELISA results showed that 17V05 could bind to IL-17A with an EC_50_ of 0.05 nM, which was the same as that of the parent mAb 608 (Figure 2(a)). We then examined its binding to VEGF, and 17V05 also showed a high affinity for VEGF with an EC_50_ value of 0.37 nM, comparable to that of D2-Fc (EC_50_ = 0.39 nM), a fusion protein with D2 fused to the N-terminus of an Fc (Figure 2(b)). In contrast, bevacizumab and 608 failed to bind to IL-17A and VEGF, respectively, indicating that the bispecific fusion protein gained the ability to bind to IL-17A and VEGF simultaneously, whereas the two mAbs did not. We confirmed the high binding capacity of 17V05 for both targets with Biacore. The equilibrium dissociation constants (KDs) of 17V05 for IL-17A and VEGF were 18.4 pM and 11 pM, respectively, on par with those of their mAb counterparts (Figure 2(c)). These data suggest that 17V05 can effectively interact with its targets without observable steric hindrance.

### 3.3. Treatment With 17V05 Potently Inhibited the Production of VEGF by RPE Cells

**Figure 3:**
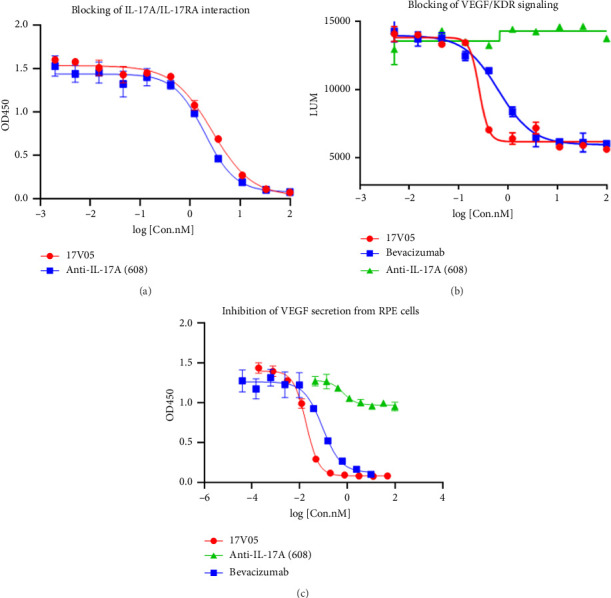
Treatment with 17V05 potently inhibited the production of VEGF by RPE cells. (a) Treatment with 17V05 blocked the interaction of IL-17A with IL-17RA as effectively as 608 in an ELISA-based assay (*n* = 3). (b) The 17V05 protein more effectively (∼3-fold) blocked the signaling of VEGF through KDR than bevacizumab in a luciferase-reporter cell-based assay. In contrast, 608 failed to interfere with the signaling (*n* = 3). (c) The 17V05 protein was 9-fold more potent than bevacizumab in inhibiting the production of VEGF by RPE cells, while 608 slightly inhibited VEGF secretion (*n* = 3). All assays were performed in duplicate. KDR: kinase insert domain receptor (VEGFR2); RPE: retinal pigment epithelium.

Given that IL-17A is able to increase the production of VEGF [19], we next examined the ability of 17V05 to inhibit VEGF secretion from RPE cells, an ocular cell line that plays key roles in pathogenic neovascularization. We first confirmed that 17V05 effectively blocked the interaction of IL-17A with IL-17RA with an IC_50_ of 2.77 nM, on par with the 1.97 nM of the anti-IL-17A parental mAb 608 (Figure 3(a)). We then showed that the D2 domains in 17V05 could effectively block the VEGF/KDR signaling pathway with a luciferase reporter system (Promega). As expected, adding 17V05 to reporter cells significantly suppressed luciferase signals with an IC_50_ value of 0.26 nM, comparable to that of the anti-VEGF parental mAb bevacizumab, whose IC_50_ was 0.64 nM (Figure 3(b)). Importantly, when treating RPE cells with 17V05, the bispecific fusion protein potently inhibited the secretion of VEGF from RPE cells with an IC_50_ value of 0.01 nM, which was 9-fold better than the 0.09 nM of bevacizumab. In contrast, 608 alone only slightly inhibited VEGF secretion from RPE with an IC_50_ value (0.68 nM) 68-fold larger than that of 17V05 and an incomplete suppression at up to 100 nM (Figure 3(c)), indicating that 17V05 has synergistic effects on the inhibition of VEGF production from RPE cells by orchestrating the blockade of IL-17A signaling and VEGF signaling.

### 3.4. Treatment With 17V05 Potently Inhibited the Production of Multiple Proinflammatory Cytokines and Chemokines by RPE Cells That Are Involved in AMD Pathogenesis

**Figure 4:**
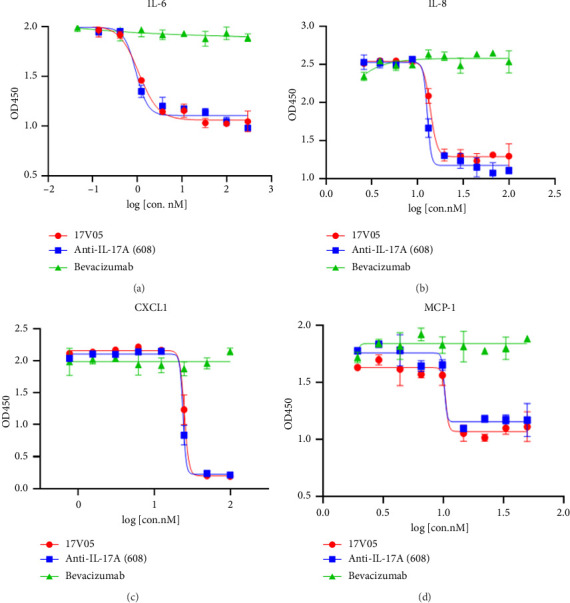
Treatment with 17V05 potently inhibited the production of multiple proinflammatory cytokines and chemokines by RPE cells. (a) Treatment with 17V05 inhibited the secretion of IL-6 from RPE cells as effectively as 608, whereas bevacizumab had no effects (*n* = 3). (b) Treatment with 17V05 inhibited the secretion of IL-8 from RPE cells as effectively as 608, whereas bevacizumab had no effects (*n* = 3). (c) The 17V05 protein inhibited the secretion of CXCL1 from RPE cells as effectively as 608, whereas bevacizumab had no effects (*n* = 3). (d) The 17V05 protein inhibited the secretion of MCP-1 from RPE cells as effectively as 608, whereas bevacizumab had no effects (*n* = 3). All assays were performed in triplicate. IL-6: interleukin-6, IL-8: interleukin-8, CXCL1: the chemokine (C-X-C motif) ligand-1, and MCP-1: monocyte chemoattractant protein-1.

Multiple inflammatory cytokines, including IL-6 and IL-8, have been shown to be involved in the development of AMD [17], and macrophages, which are recruited to the inflammatory sites by chemokines, such as CXCL1 and MCP-1, may play important roles in patients who cannot completely respond to anti-VEGF therapies [11]. Treatment of RPE cells with 17V05 effectively inhibited the secretion of IL-6 and IL-8 with IC_50_ values of 1.08 nM and 13.84 nM, respectively, comparable to the 0.93 nM and 12.81 nM of the parental counterpart 608, whereas no inhibitory effects were observed when bevacizumab was added to the cells (Figures 4(a) and 4(b)), suggesting that the production of both IL-6 and IL-8, which are downstream signaling molecules of IL-17 in eyes, can be downregulated by 17V05. Similarly, blockade of the IL-17 signaling pathway with 17V05 and 608 also effectively blocked the production of CXCL1 and MCP-1, indicating that 17V05 may reduce the recruitment of macrophages by these chemokines in eyes (Figures 4(c) and 4(d)).

### 3.5. Treatment With 17V05 Exhibited Potent Inhibitory Effects on the Development of AMD in the Laser-Induced CNV Model

**Figure 5:**
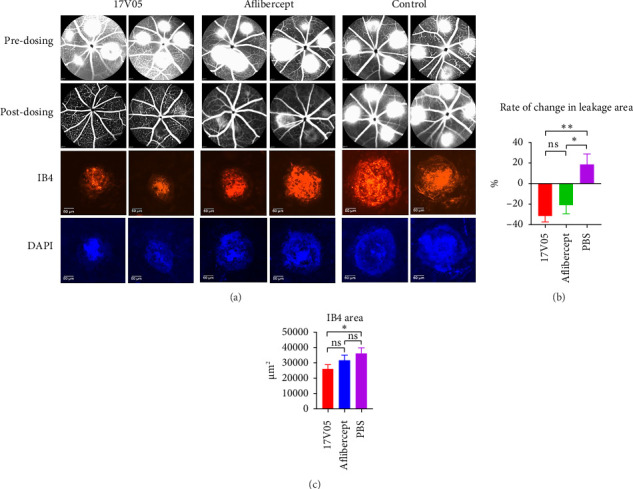
Treatment with 17V05 exhibited more potent inhibitory effects on the development of AMD than aflibercept in the laser-induced CNV model. (a) Pictures of eyes (8 mice per group, total 16 eyes per group) with laser-induced CNV before (upper panel) and 7 days after dosing with 17V05, aflibercept, and PBS (lower panel) (*n* = 2). Top two panels are FFA pictures before and after dosing, and the bottom two panels are corresponding IB4 staining (red) and DAPI staining (blue). (b) The percentage of changes in total leakage areas in the eyes of mice treated with 17V05, aflibercept, and PBS. Formula = (Leakage Areas_at day 7-after-dosing_ − Leakage Areas_at day 0-before-dosing_)/Leakage Areas_at day 0-before-dosing_ × 100. (c) Neovascular blood vessels in the retina were stained with isolectin GS-IB4 after being treated with 17V05, aflibercept, or PBS. ^∗∗^ depicts *p* < 0.01, ^∗^ depicts *p* < 0.05, and ns = not significant. IB4: isolectin GS-IB4; DAPI: 4′,6-diamidino-2-phenylindole.

Aflibercept is effective in treating VEGF-induced AMD and is the current standard of care in the clinic. Given that aflibercept is also a composite of extracellular domains of VEGFR1, including domain 2, we decided to compare the efficacy of 17V05 with aflibercept in a laser-induced CNV mouse model. Mice harboring the human IL-17A gene (humanized mice) were used in this study since the parental mAb 608 in 17V05 cannot cross react with mouse IL-17A. When 17V05 and aflibercept were intravitreally injected, 17V05 almost completely cleared the leakage in the choroid on Day 7 post-treatment, resulting in significantly reduced CNV areas (*p* < 0.01) relative to the control, whereas aflibercept effectively reduced CNV areas compared to PBS but to a lesser degree (*p* < 0.05; Figures 5(a) and 5(b)). Consistent with the above result, the mice treated with 17V05 developed significantly (*p* < 0.05) fewer newly formed blood vessels than the mice treated with PBS, whereas the mice treated with aflibercept also resulted in reduced blood vessels, but with no statistical significance (*p* > 0.05) relative to the control (Figure 5(c)), indicating that 17V05 is more potent in inhibiting neovascularization than aflibercept compared to that of the control, possibly due to the synergistic effects of combining the blockade of IL-17 and VEGF signaling.

### 3.6. The Bispecific XAB4-D2 Fusion Protein, a 17V05 Surrogate, Exhibited Durable and Potent Inhibitory Effects on the Development of Ocular Neovascularization in a Persistent RNV Rat Model

**Figure 6:**
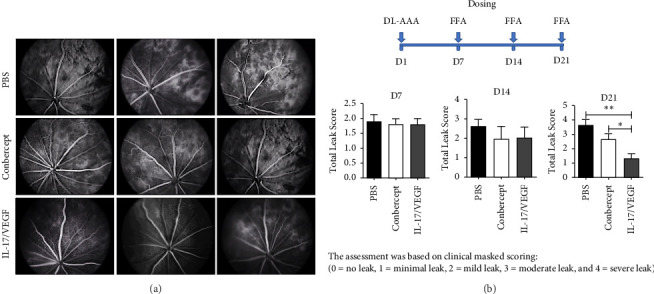
XAB4-D2, a 17V05 surrogate, exhibited durable and potent inhibitory effects on the development of ocular neovascularization in a persistent retinal neovascularization (RNV) rat model. (a) Pictures of eyes (8 rats per group) treated with PBS (upper panel), conbercept (middle panel), and XAB4-D2 (lower panel) on Day 21 post-treatment (*n* = 2). (b) Upper panel: a schematic depicts the dosing schedule and the timepoints for taking FFA. Bottom panel: the leak scores were measured on Day 14 (middle) or Day 21 (right) after treatment with PBS, conbercept, and XAB4-D2. The leak score measured on Day 7 (left) was shown as baselines. ^∗∗^ depicts *p* < 0.01; ^∗^ depicts *p* < 0.05.

Next, we adopted a newly developed long-lasting RNV model [31] to test whether a molecule with the ability to block both IL-17 and VEGF results in longer prevention of neovascularization than agents that only block VEGF pathways, since the length of treatment in the classic laser-induced CNV model usually lasts for only a week. We used XAB4-D2, a 17V05 surrogate, in which 608 was replaced with XAB4, an antibody that can interact with rat IL-17A, to examine this hypothesis with normal rats (Figure 6(a)). As expected, on Day 14 post-treatment, both 17V05 and conbercept reduced the leak areas on retinas of rat with RNV to a similar level relative to the control. Interestingly, on Day 21 post-treatment, treatment of mice with 17V05 extended the reduction of the leak areas relative to that of Day 14 and led to greater recovery compared to that of the rat treated with conbercept. In contrast, conbercept failed to sustain its inhibitory effect on neovascularization on Day 21 (Figure 6(b)). These results suggest that the bifunctional protein may have additive functions that allow the molecule to gain longer inhibitory effects relative to a VEGF-specific inhibitor.

## 4. Discussion

In this study, we devised a strategy to combine the anti-IL-17A effect with anti-VEGF function in a bispecific fusion protein to treat patients who do not respond to conventional anti-VEGF therapies. In this design, we constructed a bispecific protein trap by fusion of two copies of domain 2 of VEGFR1 to the C-terminus of a novel anti-IL-17A mAb 608, namely, 17V05. The resultant protein can effectively bind to both IL-17A and VEGF and block the signaling mediated by the two cytokines. Mounting evidence has shown that IL-17 is involved in the regulation of VEGF signaling and can regulate the secretion of VEGF from cells [24, 32]. Thus, the combination of anti-IL-17 and anti-VEGF effects may lead to a greater reduction in VEGF production than the blockade of VEGF alone. Indeed, 17V05 inhibited the secretion of VEGF from RPE cells 9-fold more effectively than bevacizumab. A similar observation was made when a luciferase reporter cell line was used to examine 17V05 activities, in which 17V05 was more potent in blocking VEGF/KDR signaling than bevacizumab. These results demonstrate that 17V05 will likely exhibit stronger inhibitory effects on VEGF-mediated neovascularization in eyes than agents that solely inhibit VEGF signaling.

Despite the fact that anti-VEGF therapy is effective in the management of various ocular diseases, 20%–60% of patients with PDA syndrome, among others, cannot completely respond to anti-VEGF therapies [11]. Patients with PDA will suffer high treatment burdens and have increased risks of long-term vision loss. The phenomenon cannot be simply explained solely by loss of the effectiveness of the anti-VEGF drugs, as multiple lines of evidence showed that only an approximately 2% rate of loss of drug efficacy was found in a large retrospective cohort of NvAMD, and the effective treatment interval of several mainstream anti-VEGF drugs on the market generally remains constant over time [33–36]. Thus, it is possible that PDA reflects the presence of other pathogenic mechanisms beyond VEGF that apparently play a dominant role in patients who are responsive to anti-VEGF therapies. After analysis of a number of clinical studies, Cousins and colleagues identified 2 distinct morphologic phenotypes of the pathologic new blood vessels in patients with AMD: one is the capillary subtype that is highly responsive to anti-VEGF therapy and rarely exhibits PDA; the other is the arteriolar subtype that is closely associated with PDA and mediates anti-VEGF resistance [11]. Angiogenesis and its associated cytokines, such as VEGF, PDGF, TGF*β* and angiopoietin-2 (Ang2), play key roles in the formation, maturation, and maintenance of the former subtype, whereas infiltrating macrophages through newly formed vessels and their induced proinflammatory cytokines play an important role in the formation of the latter subtype [11]. In addition to the proinflammatory functions of IL-17A itself, IL-17A has been shown to modulate macrophage recruitment and induce proinflammatory cytokine production in macrophages, as, reciprocally, macrophages that express IL-17 receptors can also produce IL-17 [37–41]. Thus, blockade of IL-17A may provide a promising therapeutic benefit for patients with PDA. Indeed, 17V05 effectively blocked the signaling of IL-17 through its receptor IL-17RA, inhibited the secretion of the proinflammatory cytokines IL-16 and IL8, and possibly interfered with the recruitment and functioning of infiltrating macrophages by suppressing the production of chemokines CXCL1 and MCP-1 from RPE cells. However, how these proinflammatory cytokines and chemokines play roles in recruiting macrophages to the lesions and thus contributing to the development of PDA is remained to be further elucidated.

Intravitreal injection is an invasive procedure, and repeated injection of the current standard of care, such as aflibercept, ranibizumab, or conbercept, may lead to serious complications and compromise patient compliance with the therapy [42]. The development of a long-lasting and effective therapy is urgently required for this unmet need. Two strategies were applied for this goal. First, anti-VEGF sustained-release agents are developed to provide sustained treatment. For instance, the port delivery system (PDS) with ranibizumab developed by Roche/Genentech is a nondegradable device that can be implanted in eyes and provide continuous delivery of ranibizumab for up to 6 months. Others, such as GB102 developed by GrayBug Vision, can gradually release the drug and achieve visual benefits after a single injection for 6 months with an ability to biodegrade over time [42]. Second, the construction of bispecific antibodies that simultaneously target two complementary pathways that are both involved in the pathogenesis of the diseases may offer enhanced therapeutic effects, leading to prolonged treatment intervals. Faricimab, a bispecific antibody that targets both VEGF and Ang2, exhibited comparable efficacy to ranibizumab or aflibercept with an extended treatment interval for up to 4 months in various trials, indicating that blockade of multiple angiogenesis pathways can lead to better and longer suppression of new blood vessel development than blockade of VEGF alone [42, 43]. However, Friedlander and Usui-Ouchi argued that higher potency and long-lasting anti-VEGF therapy may not necessarily be good, as potent, long-lasting VEGF antagonists may damage tissues that are dependent upon VEGF activity to survive or function [44]. In addition, although Ang2 plays important roles in vessel remodeling, maturation, and vascular permeability, little is known about its roles in the regulation of macrophages and their associated proinflammatory cytokines. Thus, whether Ang2-based bispecific antibodies are beneficial to patients with PDA remains to be further studied.

In summary, we devised a strategy to simultaneously block two distinct pathogenic pathways for the development of AMD, IL-17-induced inflammation and VEGF-mediated neovascularization. We showed that the bispecific fusion protein 17V05 effectively inhibited multiple proinflammatory cytokines and chemokines, including IL-6, IL-8, CXCL1, and MCP-1, and thus may be effective in treating immune-induced PDA. Furthermore, 17V05 was more potent in the inhibition of laser-induced CNV than aflibercept and exhibited potent and long-lasting therapeutic effects compared to conbercept in vivo. To the best of our knowledge, this is the first bispecific antibody consisting of both an anti-IL-17 agent and a VEGF targeting domain for the treatment of AMD. Thus, we provide a novel and promising strategy and provide a new choice for treating AMD patients resistant to anti-VEGF therapies.

## Figures and Tables

**Table 1:** Scoring criteria.

Grade 1	No hyperfluorescence
Grade 2	Hyperfluorescence with no leakage
Grade 3	Early hyperfluorescence with late leakage
Grade 4	Bright early hyperfluorescence with late leakage beyond borders
Grade 3 and 4	Lesions were considered “leaking lesions”

## Data Availability

The datasets used and/or analyzed during the current study are available from the corresponding author on reasonable request.
